# Author Correction: Genome‐wide mapping of plasma protein QTLs identifies putatively causal genes and pathways for cardiovascular disease

**DOI:** 10.1038/s41467-018-06231-z

**Published:** 2018-09-18

**Authors:** Chen Yao, George Chen, Ci Song, Joshua Keefe, Michael Mendelson, Tianxiao Huan, Benjamin B. Sun, Annika Laser, Joseph C. Maranville, Hongsheng Wu, Jennifer E. Ho, Paul Courchesne, Asya Lyass, Martin G. Larson, Christian Gieger, Johannes Graumann, Andrew D. Johnson, John Danesh, Heiko Runz, Shih-Jen Hwang, Chunyu Liu, Adam S. Butterworth, Karsten Suhre, Daniel Levy

**Affiliations:** 1 Framingham Heart Study, Framingham, 01702 MA USA; 2 Population Sciences Branch, Division of Intramural Research, National Heart, Lung, and Blood Institute, National Institutes of Health, Bethesda, 20892 MD USA; 3 Department of Medical Sciences, Uppsala University, 75105 Uppsala, Sweden; 4 Department of Immunology, Genetics and Pathology, Uppsala University, 75105 Uppsala, Sweden; 5 Department of Cardiology, Boston Children’s Hospital, Boston, 02115 MA USA; 6 MRC/BHF Cardiovascular Epidemiology Unit, Department of Public Health and Primary Care, University of Cambridge, Cambridge, CB1 8RN UK; 7 Research Unit of Molecular Epidemiology, Helmholtz Zentrum München, German Research Center for Environmental Health, Ingolstädter Landstraße 1, 85764 Neuherberg, Germany; 8 Institute of Epidemiology II, Helmholtz Zentrum München, German Research Center for Environmental Health, Ingolstädter Landstraße 1, 85764 Neuherberg, Germany; 9 MRL, Merck & Co., Inc, Kenilworth, 07033 NJ USA; 10 Computer Science and Networking, Wentworth Institute of Technology, Boston, 02115 MA USA; 11 Cardiovascular Research Center and Division of Cardiology, Department of Medicine, Massachusetts General Hospital, Boston, 02114 MA USA; 12 Department of Mathematics and Statistics, Boston University, Boston, 02115 MA USA; 13 Department of Biostatistics, Boston University School of Public Health, Boston, 02118 MA USA; 14 German Center for Diabetes Research (DZD), Ingolstädter Landstraße 1, 85764 Neuherberg, Germany; 15 Scientific Service Group Biomolecular Mass Spectrometry, Max Planck Institute for Heart and Lung Research, W.G. Kerckhoff Institute, Ludwigstr. 43, D-61231 Bad Nauheim, Germany; 16 British Heart Foundation Cambridge Centre of Excellence, Division of Cardiovascular Medicine, Addenbrooke’s Hospital, Cambridge, CB2 0QQ UK; 17 Department of Human Genetics, Wellcome Trust Sanger Institute, Wellcome Trust Genome Campus, Hinxton, Cambridge CB10 1RQ UK; 18 NIHR Blood and Transplant Research Unit in Donor Health and Genomics, Department of Public Health and Primary Care, University of Cambridge, Cambridge, CB1 8RN UK; 19 Department of Physiology and Biophysics, Weill Cornell Medicine-Qatar, Education City, PO 24144 Doha, Qatar

Correction to: *Nature Communications*; 10.1038/s41467-018-05512-x; published online 15 August 2018.

In the originally published version of this article, financial support was not fully acknowledged. The sentence “KS was supported by the ‘Biomedical Research Program’ funds at Weill Cornell Medicine in Qatar, a program funded by the Qatar Foundation” has been added to the acknowledgement section in both the PDF and HTML versions of the article.

