# Supplementary material for: Genome‐wide mapping of plasma protein QTLs identifies putatively causal genes and pathways for cardiovascular disease
Source: Nat Commun. 2018 Aug 15;9:3268. doi: 10.1038/s41467-018-05512-x (PMC6093935; doi:10.1038/s41467-018-05512-x)
Supplement: Supplementary file 17 — Supplementary Information [file 41467_2018_5512_MOESM17_ESM.pdf]

Genome-wide Mapping of Plasma Protein QTLs Identifies Putatively Causal Genes and Pathways for Cardiovascular Disease

Chen et al.

Supplementary Information

Supplementary Note 1

Framingham Heart Study: We thank all the study participants who helped to create this valuable resource and supported this work. We thank the data management group of FHS for organizing and providing these data. We thank the National Institutes of Health Fellows Editorial Board members for their valuable edits and comments. This study used the high-performance computational capabilities of the Biowulf Linux cluster at the National Institutes of Health, Bethesda, MD. KORA: KS was supported by 'Biomedical Research Program' funds at Weill Cornell Medicine in Qatar, a program funded by the Qatar Foundation. We gratefully acknowledge the contribution of all members of field staff conducting the KORA F4 study. Most of all, we thank all study participants for their invaluable contributions to this study. INTERVAL: We acknowledge the participation of all INTERVAL volunteers. We thank the INTERVAL study co-ordination teams (at the Universities of Cambridge and Oxford and at NHS Blood and Transplant [NHSBT]), including the blood donation staff at the 25 static centers, for their help with INTERVAL participant recruitment and study fieldwork, as well as the Cambridge BioResource and NHSBT staff for their help with volunteer recruitment. We thank the INTERVAL Operations Team headed by Dr Richard Houghton and Dr Carmel Moore, and the INTERVAL Data Management Team headed by Dr Matthew Walker. We thank all the staff at SomaLogic for processing and running the proteomic assays.

Supplementary Figure 1. Effect size of cis-pQTL rs941590 on SERPINA10 concentration.
A missense variant, rs941590 explained 32% of inter-individual variation in SERPINA10 levels.
Circle denotes mean value of SERPINA10 concentration (pg/mL) for each genotype.

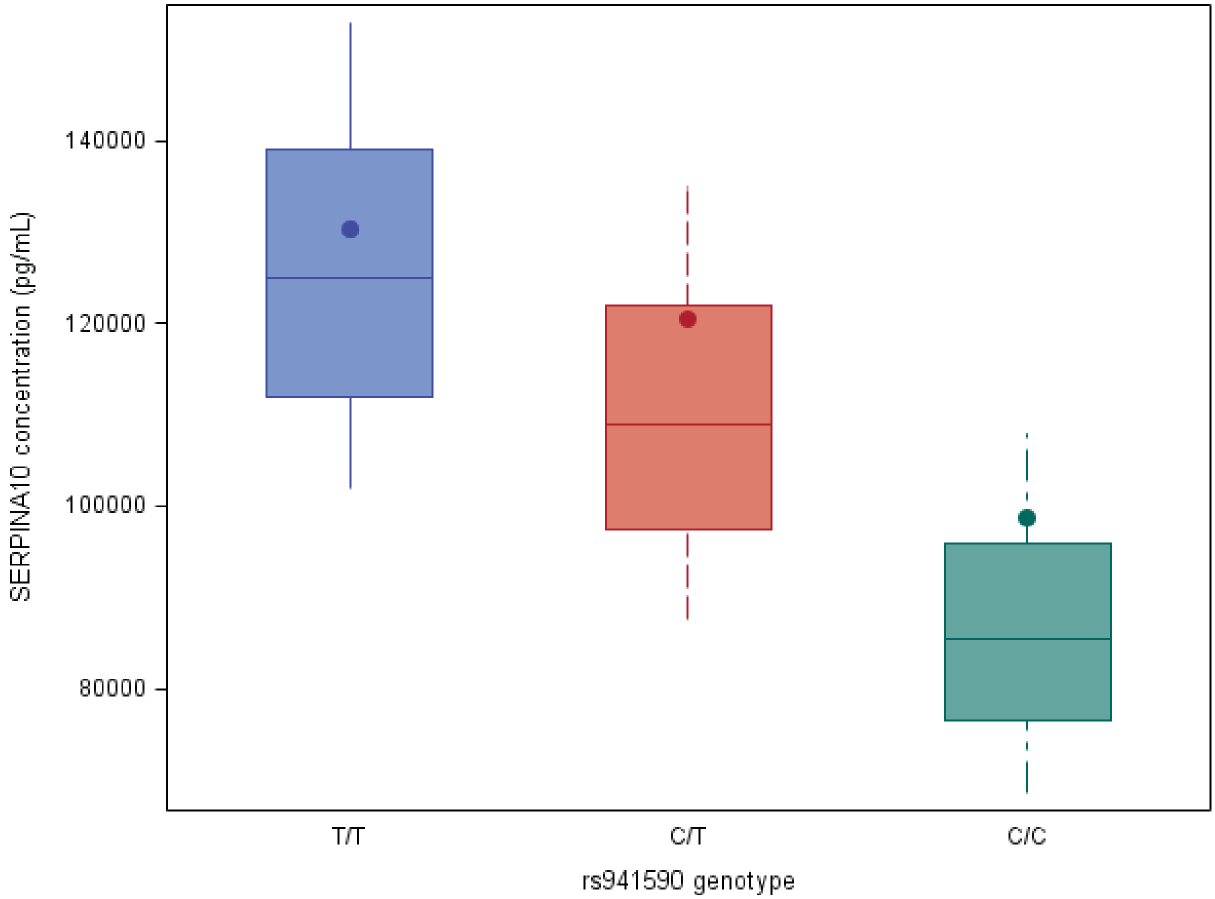

Supplementary Figure 2. *Trans*-pQTL variants regulate circulating protein levels through the expression of nearby *cis*-eGenes

Figure 2a

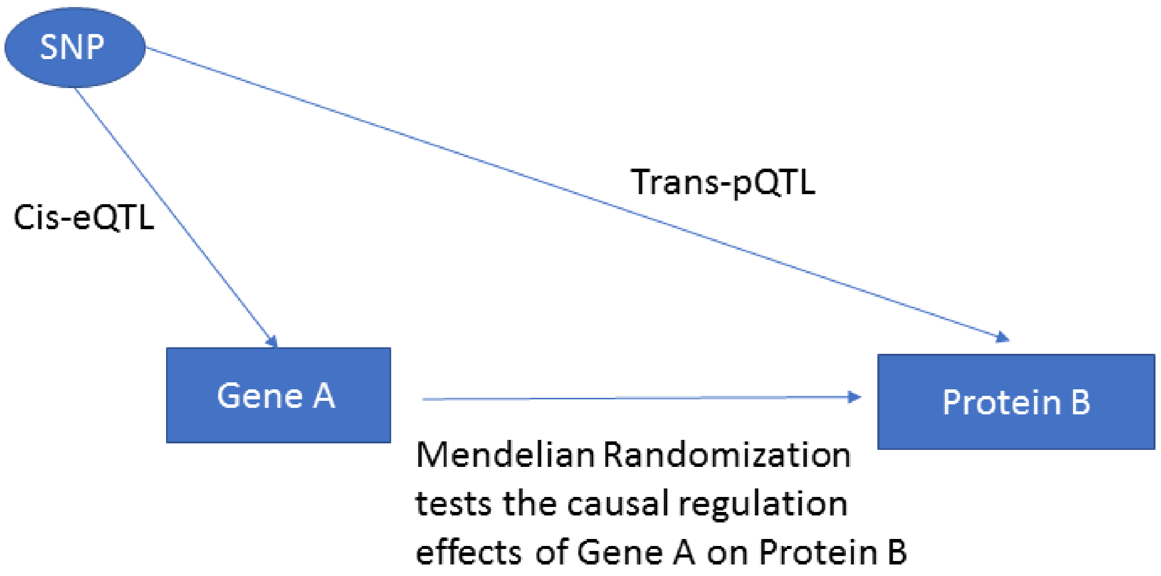

Figure 2a: *Trans*-pQTL variants regulate circulating protein levels through the expression of nearby *cis*-eGenes: a pQTL variant (SNP) is a *cis*-eQTL for Gene A (*Cis*-eGene) and a *trans*-pQTL for Protein B (Protein). Mendelian randomization establishes a causal effect of Gene A expression on circulating Protein B levels.

Figure 2b

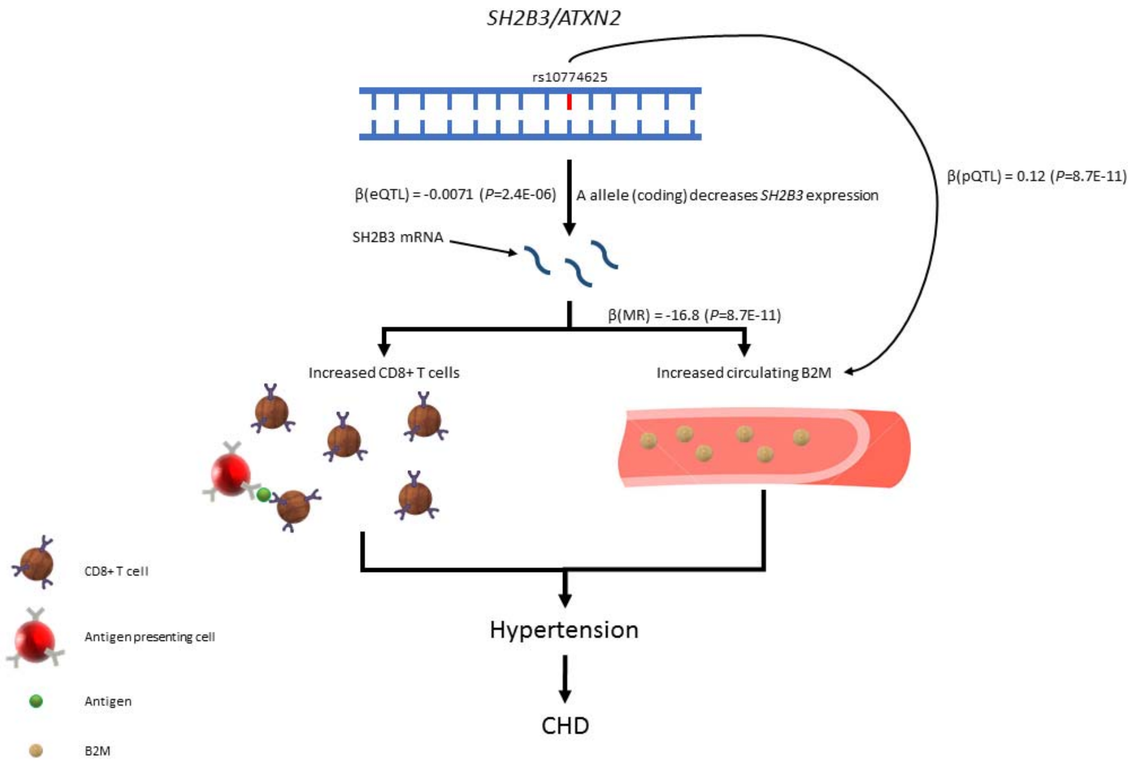

Figure 2b: Example of circulating B2M levels regulated by *SH2B3* expression. The A allele of rs10774625 (*ATXN2/SH2B3 locus*) reduces SH2B3 expression, which in turn increases CD8+ T cell differentiation[1] and circulating B2M levels[2]. The resulting increases in CD8+ T cells and plasma B2M levels result in a greater risk of hypertension[3,4].

Supplementary References:

1 Dale, B. L. & Madhur, M. S. Linking inflammation and hypertension via LNK/SH2B3. *Curr Opin Nephrol Hypertens* **25**, 87-93, doi:10.1097/MNH.0000000000000196 (2016).

2 Huan, T. *et al.* Integrative network analysis reveals molecular mechanisms of blood pressure regulation. *Mol Syst Biol* **11**, 799, doi:10.15252/msb.20145399 (2015).

2 You, L. *et al.* High levels of serum beta2-microglobulin predict severity of coronary artery disease. *BMC Cardiovasc Disord* **17**, 71, doi:10.1186/s12872-017-0502-9 (2017).

4 Itani, H. A. *et al.* Activation of Human T Cells in Hypertension: Studies of Humanized Mice and Hypertensive Humans. *Hypertension* **68**, 123-132, doi:10.1161/HYPERTENSIONAHA.116.07237 (2016).
