# Supplementary material for: Genome‐wide mapping of plasma protein QTLs identifies putatively causal genes and pathways for cardiovascular disease
Source: Nat Commun. 2018 Aug 15;9:3268. doi: 10.1038/s41467-018-05512-x (PMC6093935; doi:10.1038/s41467-018-05512-x)
Supplement: Supplementary file 18 — Description of Additional Supplementary Files [file 41467_2018_5512_MOESM18_ESM.pdf]

Supplementary Data 1

Description: Protein biomarker assay performance metrics and inclusion criteria

Candidate protein biomarkers were selected a priori based on prior evidence of association with atherosclerotic CVD or its risk factors using the following complementary approaches: a) comprehensive literature search, b) proteomics discovery via mass spectrometry in the FHS or elsewhere, and c) targeting proteins coded by genes identified via gene expression profiling studies or GWAS of atherosclerotic CVD and its risk factors. Plasma protein biomarkers were assayed using a modified enzyme-linked immunosorbent assay sandwich method, multiplexed on a Luminex xMAP platform (Luminex, Inc., Austin, TX). The 'High' and 'Low' spike controls (QC1 and QC2 respectively) were used to calculate intra- and inter-assay coefficients of variation (CV) for each protein. A total of 14 proteins had low call-rate (<90%) mainly due to values falling below the lower detection limit that were excluded for the current study.

Supplementary Data 2

Description: Clinical characteristics of discovery sample

All FHS participants underwent periodic clinical examinations with standard protocols. A three-physician panel performed weekly medical chart reviews.

Supplementary Data 3

Description: pQTLs with reference SNP cluser IDs from 1000-Genomes-imputed GWAS and Exome Chip variants using hg build 37

Linear mixed-effects models were used to test for associations between inverse-rank normalized protein levels and genetic variants from 1000G or Exome Chip using an additive genetic model. Cis: variant within 1Mb (upstream or downstream) of the protein coding gene; Trans: variant >1Mb from the protein coding gene. EAF: effect allele frequency.

Supplementary Data 4

Description: Required effect size (beta) to reach 80% of power in the FHS discovery sample

An additive genetic model with no gene-gene interactions, and a population mean=0 and standard deviation=1 for all rank-normalized protein levels was used for power calculation. The estimated effect size is the standard deviation change in rank-normalized protein values per allele increase.

Supplementary Data 5

Description: Pruned pQTL SNPs (LD $r^2<0.1$) from 1000-Genomes-imputed GWAS

Linkage disequilibrium (LD) was computed as the square of Pearson's correlation (r^2) between imputed additive dosages of genotypic variants within the same chromosome across 8,481 FHS individuals with genotype data. Pruned pQTLs for a given protein were defined as those with LD $r^2<0.2$ with other pQTLs at a genomic locus.

Supplementary Data 6

Description: Summary of Sentinel pQTL loci

For a genetic locus with multiple pQTLs in LD (i.e., LD $r^2>0.2$), the pQTL with the lowest P value was selected as the sentinel pQTL for that locus.

Supplementary Data 7

Description: pQTL variants in protein coding regions

All exonic pQTL variants were annotated by the Ensemble database. cdna_pos: SNP position on cdna, if the predicted function is coding, 3'UTR or 5'UTR;cds_pos: SNP position on cds, if the predicted function is coding; aa_pos: position of the first amino acid (possibly) effected in the resultant peptide chain, if the predicted function is coding; aa_change: Peptide <reference amino acid(s),">", observed amino acid(s)_1 [,"|", observed amino acid(s)_2, ...] > ; syn: synonymous; nonsyn: non-synonymous.

Supplementary Data 8

Description: Rare pQTL variants from Exome Chip

Variants with minor allele frequency <1% genotyped on Exome Chip.

Supplementary Data 9

Description: Insertion/deletion polymorphisms

Insertion and deletion polymorphism from 1000-Genomes-imputed GWAS. R: reference; D: deletion; I: insertion.

Supplementary Data 10

Description: Replication of sentinel pQTLs in external cohorts and previous GWAS

103 sentinel pQTLs were independently measured in the INTERVAL and KORA studies. Reported P values are nominal P values from each cohort, unadjusted for multiple correction. Resampling counts: we performed pQTL analyses with 1,000 resamplings of 3,300 FHS participants (sample size of approximately 3300 in INTERVAL). We counted the number of results with P<0.05/n in the 1,000 resamplings, where n is the number of pQTL variants that were tested for replication in INTERVAL.

Supplementary Data 11

Description: Overlap of pQTL variants from 1000-Genomes-based GWAS with eQTLs

List of eQTLs variants (genetic variants associated with whole blood gene expression levels in FHS participants) from FHS eQTLs database that overlap with pQTLs reported here. Only the non-redundant pQTL variants for each locus is listed.

Supplementary Data 12

Description: Proteins with >75% probability of colocalization with eQTLs

Colocalization analysis for genes within 1 Mb region (upstream and downstream) of each sentinel cis-pQTL variant. PP.H1 (causal variant for gene expression only), PP.H2 (causal variant for protein only), PP.H3 (two distinct causal variants), PP.H4 (one common causal variant).

Supplementary Data 13

Description: pQTL variants that coincide with coronary heart disease GWAS SNPs

List of CHD GWAS SNPs from the CARDIoGRAMplusC4D Consortium that coincide with pQTLs reported here. SNP-protein association was calculated using FHS data; SNP-CHD association abstracted from published CARDIoGRAMplusC4D Consortium GWAS.

Supplementary Data 14

Description: Mendelian randomization for expression of *cis*-eGenes regulating proteins in *trans*

Mendelian randomization (MR) was conducted using the expression of all genes within 1 Mb of the trans-pQTL locus as the exposure, cis-eQTLs associated with these genes (from the FHS whole blood gene expression database) as instrumental variables and circulating protein levels as the outcome. P values were calculated from the MR Wald ratio test.

Supplementary Data 15

Description: Mendelian randomization test results for protein associations with coronary heart disease risk

Mendelian randomization (MR) was conducted using circulating protein levels as the exposure, pruned cis-pQTLs (LD $r^2<0.1$*) for each protein as instrumental variables (IVs), and CHD as the outcome. P values were calculated from the Wald ratio test (single IV) or inverse-variance weighted estimates (multiple IVs). Causal effect estimates of proteins on CHD are reported per standard error increment in inverse-rank normalized protein level.*
